# Metacarpal Lengthening Of The Thumb Via Distraction Osteogenesis: A Case Report

**DOI:** 10.5704/MOJ.1503.009

**Published:** 2015-03

**Authors:** WH Chung, CK Eo, Z Muspirah, A Sood

**Affiliations:** Department of Orthopaedics, Hospital Sultanah Bahiyah, Alor Setar, Malaysia; * Department of Orthopaedics, Hospital Kulim, Kulim, Malaysia

**Keywords:** Thumb amputation, Metacarpal lengthening, Distraction Osteogenesis, Callotasis

## Abstract

Amputation of the thumb invariably causes marked functional impairment of the hand especially, pinch and grasp functions. In rural areas where highly skilled microvascular surgeries are not available, distraction osteogenesis provides an easy and safe alternative of thumb reconstruction. We report a case of crush injury of the right hand in a 37 year old gentleman in which the right thumb was amputated at the level of the proximal phalanx. Metacarpal lengthening of the thumb was performed by using distraction osteogenesis.

## Introduction

The thumb contributes 40% of the hand function. Thus, thumb amputation causes marked functional impairment of the hand especially, pinch and grasp motions. An amputated thumb should be replanted preferably. If replantation is not possible, several methods of thumb reconstruction are implied such as distraction osteogenesis, osteoplastic reconstruction, pollicization of fingers, toe-to-thumb transfer and thumb prosthesis^1^.

Distraction osteogenesis or callotasis involves bone lengthening by osteotomy, slow distraction of healing fracture callus and stabilization with an external fixator^2,3^. There are three phases in callotasis, which are latency, activation and consolidation. Latency is the phase following osteotomy and application of the distractor and usually ranges within 1 to 7 days. Activation phase involves gradual distraction by the distractor and, finally followed by consolidation phase when the desired length is achieved^3^.

We report a case of metacarpal lengthening of the thumb by distraction osteogenesis after a traumatic amputation of the thumb.

## Case report

A 37 year old gentleman who alleged an industrial accident sustained a crush injury to his right hand. There were traumatic amputations to his right thumb, index, middle and ring fingers which were not feasible for replantation. The amputations were through the proximal phalanx. Three months after the injury, we decided to lengthen the first metacarpal via callotasis to improve the pinch function of the hand. Informed consent was obtained from the patient. Lengthening of the second metacarpal was not accomplished as it was disagreed by the patient.

Before surgery, detailed information regarding the procedure was conveyed to the patient. The operation was done under general anaesthesia and a pneumatic tourniquet was applied to the arm. A small nick was made over the skin. The bone was drilled bicortically with a 1.5 mm drill bit at right angle to the bone. Two pairs of 2.0 mm threaded half-pins were inserted parallel to one another. Distractor was applied. A 1.5 cm longitudinal dorsoradial incision was made over the metacarpal. Dissection was made down to the bone and the periosteum was incised longitudinally between the two pairs of the pins. The periosteum was elevated carefully. Caution was taken to avoid injury of the extensor tendons. Subperiosteal osteotomy was done at the diaphyseal region with multiple drill holes using a 2.0 mm drill bit. An osteotome was not needed. The osteotomy was distracted to ensure complete osteotomy and was closed again so that both bone ends were in contact (Figure 1a). The periosteum was closed with absorbable sutures. The alignment was confirmed with image intensifier.

**Fig. 1a:**
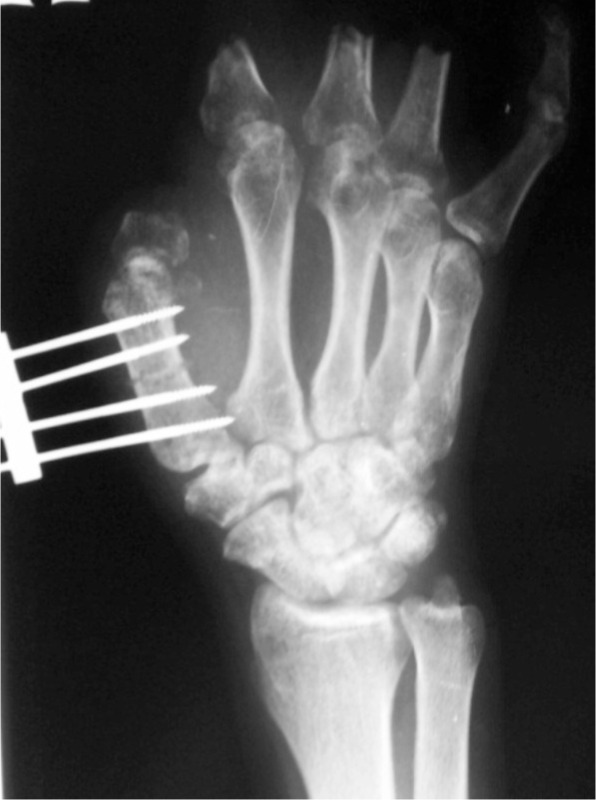
Day 1 post mini distractor and osteotomy.

Lengthening was started 5 days post-operatively, two daily lengthenings of 0.5 mm each, giving 1.0 mm per day. The patient was guided on the distraction procedures and first few distractions by the patient were done under supervision. Fortnightly follow-ups were given during which the efficacy of the distraction, stability of the distractor and pin site care were assessed (Figure 1b). Active finger exercises began during the early post-operative period. Distraction was discontinued 30 days post-operatively after the desired length was achieved. The consolidation phase took about 90 days (Figure 1c). The distractor was removed when there was cortical continuity of minimum three cortices and intramedullary bone formation radiologically. In the same setting, the first webspace was deepened by performing a four-flap Z-plasty. The thumb musculature was incised slightly to gain more length.

**Fig. 1b:**
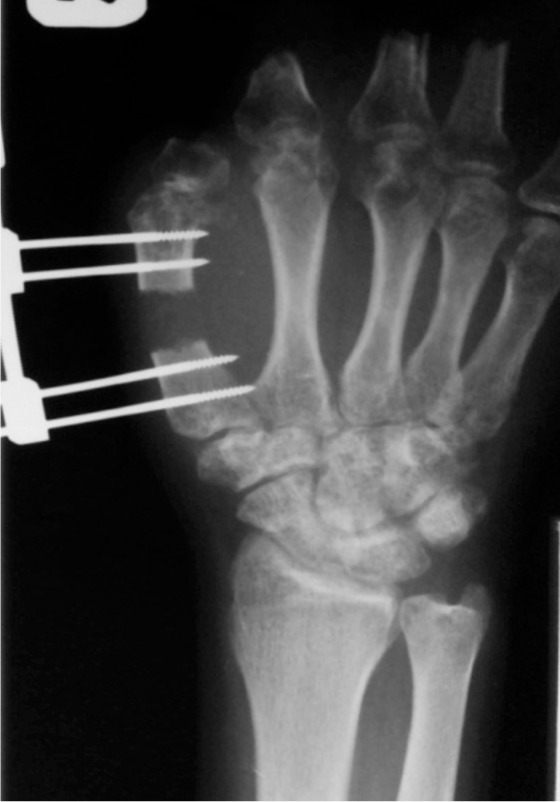
2 weeks of metacarpal lengthening.

**Fig. 1c:**
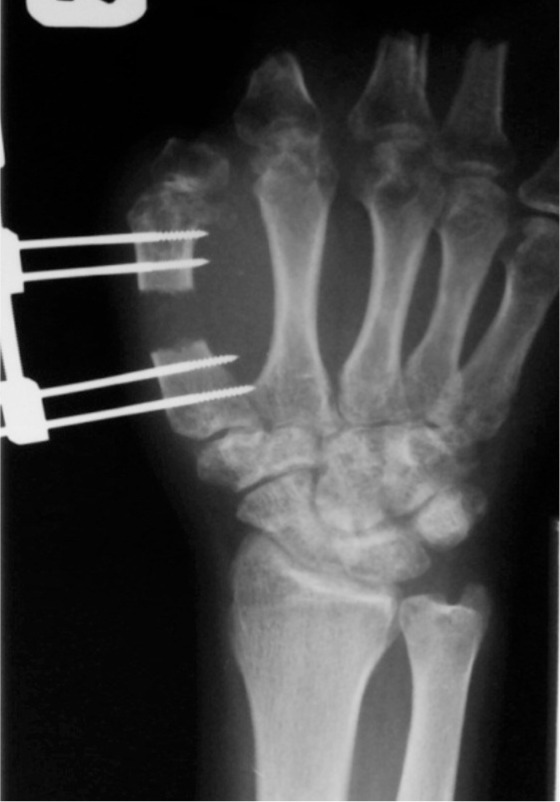
25 mm lengthening or 60% of the preoperative bone length was achieved. Consolidation phase showed good callus formation.

**Fig. 2:**
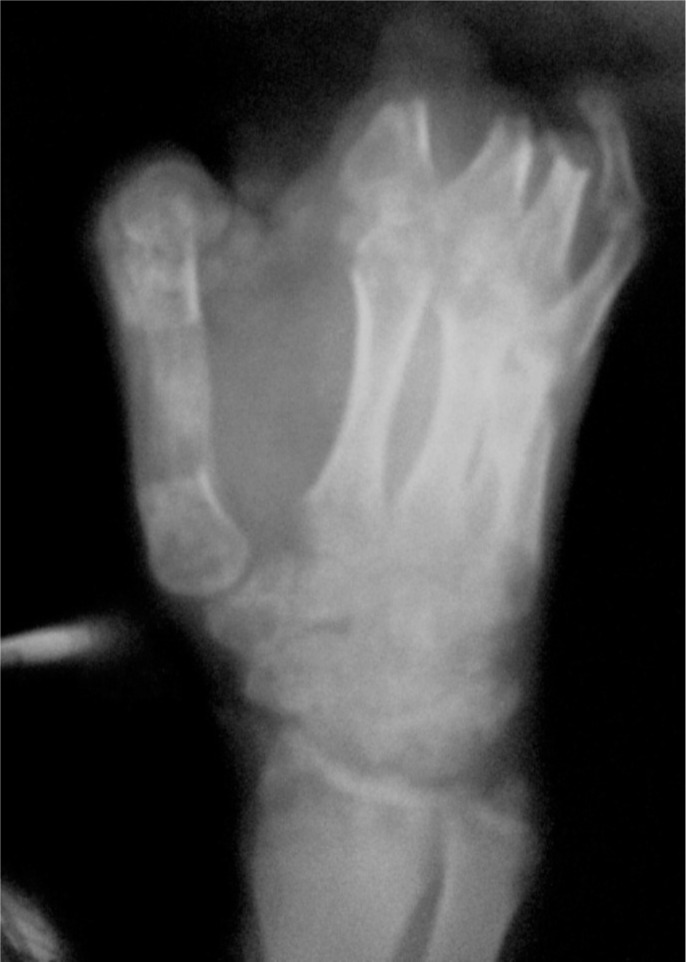
Distractor was removed when there was radiological evidence of cortical continuity and intramedullary bone formation.

**Fig. 3:**
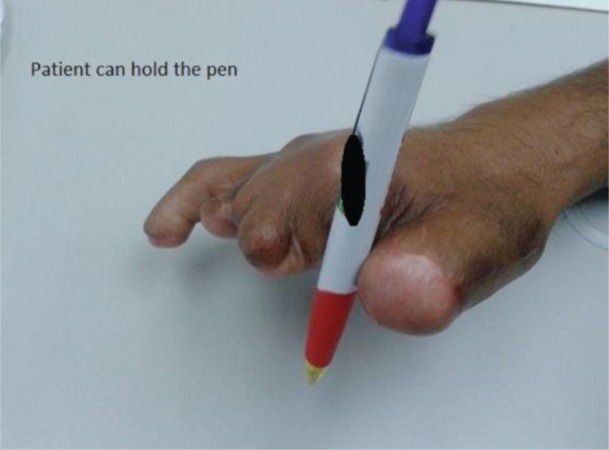
Final outcome at 5 months post-operative period. Pinch function was improved. Patient was able to write, hold a spoon and button his clothes.

Lengthening of 25 mm or 60% of the pre-operative bone length was achieved (Figure 2). Pinch function was improved. The patient was able to hold a pen and write, hold a spoon and button his clothes (Figure 3). No complications such as pin tract infection, pin loosening, bowing or angulation of the lengthened bone, premature consolidation, callus fracture or sensory loss were observed. However, we were not able to assess the strength and endurance as the patient has returned back to his country due to logistic reasons.

## Discussion

Various techniques such as osteotomy and intercalary bone grafting have been described in treating short metacarpals. Acute lengthening poses various disadvantages such as inadequate lengthening, morbidity to the donor site and neurovascular compromise. By gradual distraction, greater bone length can be achieved and fewer complications are observed^3^. Distraction lengthening of the thumb was first described by Matev in 1970. In 2003, he reported his experience with 92 thumb metacarpal lengthenings^4^.

Possible complications have been reported in distraction osteogenesis such as pin tract infection, pin loosening, bowing of the lengthened bone, premature consolidation, callus fracture and non-union^2,3^. However, we did not experience any of the major complications described.

On the other hand, Matev reported that bone grafting with iliac crest was required to bridge the interfragmentary space in one third of his adult patients. In our case, bone graft was not used. The distractor was kept until the fracture consolidated, as suggested by Finsen. Although the distractor remained for a longer period, there was no sign of infection. This might be attributable to proper pin tract care and good patient’s compliance.

According to F Khan, in order to prevent complications such as stiffness, angulation and subluxation of the metacarpophalangeal joint, lengthening should not be more than 40% or 20 mm of the pre-operative bone length. In our case, 25 mm or 60% of the pre-operative bone length was achieved, yet such complications were not observed. Preoperatively, the thenar musculature and the carpometacarpal joint mobility were preserved and this is crucial in order to maintain the thumb alignment in relation to the fingers during the lengthening process. However, the muscle action of abduction, adduction and opposition were not addressed specifically but over a period of time, the patient was at least able to use the thumb for some basic hand functions.

There was no consensus regarding the optimal rhythm and frequency of lengthening. Most authors recommended lengthening between 0.5 mm to 1.0 mm per day; two to four daily lengthening^2,3,5^. In our case, two daily lengthenings of 0.5 mm each giving 1.0 mm per day was appropriate to us as no complications were noted.

Osteotomy is usually done at the metaphyseal-diaphyseal region as it has good blood supply and large surface area. In our case, osteotomy with multiple drill holes was performed at the diaphysis. Good callus formation and subsequent consolidation without bone graft were observed. We believe that stability of the fixation and preservation of the periosteum at the osteotomy site might play a more important role in bone healing.

In conclusion, distraction osteogenesis is safe and easy. It can be done in district hospitals where microsurgeries are not available. We recommend that stability of the fixation, preservation of the periosteum, pin tract care and patient’s education and compliance should be emphasized. Presently, only lengthening of the thumb was attempted. We think that, in future, a special device could be created in which the distal fragment may be rotated gradually or in a few acute episodes to further improve the thumb function.
